# Translating community-based participatory research into broadscale sociopolitical change: insights from a coalition of women firefighters, scientists, and environmental health advocates

**DOI:** 10.1186/s12940-023-01005-7

**Published:** 2023-08-30

**Authors:** Jennifer Liss Ohayon, Sharima Rasanayagam, Ruthann A Rudel, Sharyle Patton, Heather Buren, Tony Stefani, Jessica Trowbridge, Cassidy Clarity, Julia Green Brody, Rachel Morello-Frosch

**Affiliations:** 1 Silent Spring Institute, 320 Nevada Street, Suite 302, Newton, MA 02460 USA; 2 California Breast Cancer Research Program, University of California, Office of the President, Oakland, CA USA; 3 Commonweal Biomonitoring Resource Center, Bolinas, CA USA; 4 United Fire Service Women, San Francisco, CA USA; 5 San Francisco Firefighters Cancer Prevention Foundation, San Francisco, CA USA; 6 School of Public Health, University of California, Berkeley, Berkeley, CA USA; 7 Department of Environmental Science, Policy, and Management, University of California, Berkeley, Berkeley, CA USA

**Keywords:** Community-based participatory research, CBPR, Firefighters, Breast cancer, Biomonitoring, Occupational health

## Abstract

**Background:**

We report on community-based participatory research (CBPR) initiated by women firefighters in order to share successful elements that can be instructive for other community-engaged research. This CBPR initiative, known as the Women Worker Biomonitoring Collaborative (WWBC) is the first we are aware of to investigate links between occupational exposures and health outcomes, including breast cancer, for a cohort of exclusively women firefighters.

**Methods:**

In order to be reflective of the experiences and knowledge of those most intimately involved, this article is co-authored by leaders of the research initiative. We collected leaders’ input via recorded meeting sessions, emails, and a shared online document. We also conducted interviews (N = 10) with key research participants and community leaders to include additional perspectives.

**Results:**

Factors contributing to the initiative’s success in enacting broadscale social change and advancing scientific knowledge include (1) forming a diverse coalition of impacted community leaders, labor unions, scientists, and advocacy organizations, (2) focusing on impacts at multiple scales of action and nurturing different, yet mutually supportive, goals among partners, (3) adopting innovative communication strategies for study participants, research partners, and the broader community, (4) cultivating a prevention-based ethos in the scientific research, including taking early action to reduce community exposures based on existing evidence of harm, and (5) emphasizing co-learning through all the study stages. Furthermore, we discuss external factors that contribute to success, including funding programs that elevate scientist-community-advocacy partnerships and allow flexibility to respond to emerging science-policy opportunities, as well as institutional structures responsive to worker concerns.

**Conclusions:**

While WWBC shares characteristics with other successful CBPR partnerships, it also advances approaches that increase the ability for CBPR to translate into change at multiple levels. This includes incorporating partners with particular skills and resources beyond the traditional researcher-community partnerships that are the focus of much CBPR practice and scholarly attention, and designing studies so they support community action in the initial stages of research. Moreover, we emphasize external structural factors that can be critical for CBPR success. This demonstrates the importance of critically examining and advocating for institutional factors that better support this research.

## Background

About a decade ago, women firefighters in San Francisco began to organize around concerns about breast cancer in their ranks. San Francisco has the highest proportion of women firefighters in the U.S. as a result of successful legal action around discriminatory hiring practices [1]. Reflecting on personal experience with the illness or supporting colleagues diagnosed with breast cancer, they questioned why so many young and fit women firefighters were afflicted, how they could get appropriate action from their department and officials, and what could be done to prevent the burden of breast cancer in the first place.

These questions led firefighters to initiate a community-based participatory research (CBPR) project to investigate occupational exposures with potential relevance for breast cancer risk. In CBPR, community partners and researchers work in close collaboration on all research stages, including formulating the research questions and study design, collecting data, interpreting findings, and translating results into action. CBPR aims to elevate community expertise, generate co-owned data, increase the responsiveness of research agendas to community-identified needs, inform local organizing efforts, and strengthen environmental and health policy [2–4].

While women firefighters initiated a CBPR study in response to knowledge gaps about the links between firefighter exposures and female breast cancer, earlier studies demonstrated firefighters have higher rates of many types of cancers compared to the general population [5–11]. Other studies have shown elevated exposures among firefighters to chemicals linked to cancer and other adverse health outcomes, including exposures to per- and polyfluoroalkyl substances (PFAS), flame retardants, polycyclic aromatic hydrocarbons, dioxins, diesel exhaust, and benzene, with exposure occurring through fire suppression activities, equipment (e.g., firefighting foams and protective gear), fire stations, and vehicular emissions [12–20]. These studies, however, focus almost exclusively on men [21, 22], which is problematic given that the reported exposures include chemicals with relevance to breast cancer [23–25]. To our knowledge, the WWBC is the only study to investigate chemical exposures in a female cohort of firefighters.

The study that San Francisco women firefighters initiated in 2012 would evolve into the Women Workers Biomonitoring Collaborative (WWBC), an extended partnership aimed at understanding and responding to occupational exposures that put women workers at risk. As reflected in the name, WWBC adopted a biomonitoring approach to understand women firefighters’ exposures to chemicals of concern. Biomonitoring measures personal exposures to chemicals and their metabolites in biological matrices such as blood, urine, and hair.

## Methods

This article is co-authored by firefighters, scientists, and advocates who launched and participated in WWBC. This authorship approach allows us to document the history, outcomes, themes, and lessons of this unique CBPR partnership through the contributions of those most intimately involved. Authors met twice to discuss the structure and content of the paper in recorded sessions, with extensive input also occurring through a shared online document and emails between September 2020 and December 2021. In September 2020, ten additional semi-structured interviews were conducted by the first author with WWBC leaders, seven of whom are not authors on this paper, to add additional perspectives. These interviewees included six firefighters, three scientists, and one environmental health advocate. Interview questions covered participants’ experiences, as well as goals, benefits, and challenges of the partnership (interview questions available upon request). Interviews were recorded, transcribed, and analyzed by the first author. In addition, documents and other resources, including policy briefs and factsheets, advocacy support letters for bills, and firefighter training materials, were analyzed to investigate the partnership’s broad impacts on environmental policy and public knowledge.

## Results

**Fig. 1:**
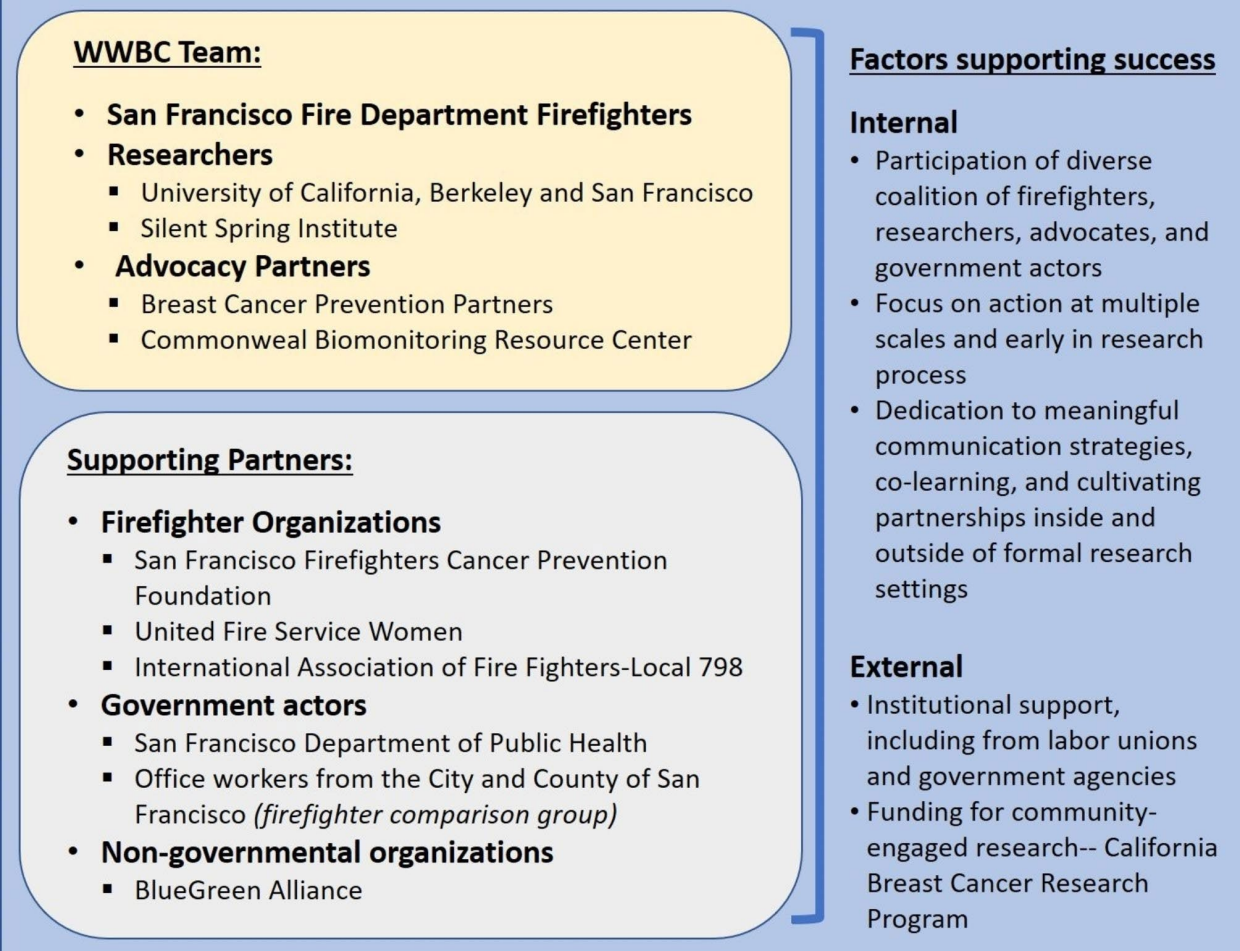
WWBC team, supporting partners, and the internal and external factors contributing to success of the project

WWBC exemplifies the CBPR approach and extends it in various ways. In particular, WWBC demonstrates how to build a coalition of partners that is more diverse than the traditional researcher-community partnerships that are the focus of much CBPR practice and scholarly attention. WWBC is characterized by a partnership among multi-disciplinary, multi-institutional scientists, individual workers, local and international unions, and environmental health and breast cancer advocacy groups. As a result of this diverse coalition, WWBC has been able to enact positive changes at multiple levels, including in occupational, research, and policy settings, while advancing science on worker exposures of concern for breast cancer. In this section, we discuss how this CBPR project was created and developed, and go into detail on participating actors and factors that contributed to its success (Fig. 1).

### The creation of a community-based participatory research project, and key scientific findings

WWBC was initiated by firefighters at the San Francisco Fire Department (SFFD) deeply concerned about the number of their colleagues diagnosed with premenopausal breast cancer. Their observations reflected San Francisco’s history as one of the first cities to actively recruit women firefighters. In response to racial and gender discrimination in hiring practices, a 1988 court order called for a force made up of 40% people of color and 10% women [26]. While women make up approximately 4% of U.S. firefighters, the proportion of women firefighters in San Francisco is 15%, with both the current and prior fire chiefs being women [27, 28]. Several firefighters who helped establish and lead the WWBC came into SFFD through the consent decree, and thus have the experience of rising in rank in a male-dominated department. The progress that San Francisco made in diversifying its workforce made it possible to have the first health study focused on women firefighters. While the health concerns among this population were fueled primarily by breast cancer diagnoses in their ranks, attention to the issue was aided by increasing media coverage [29, 30] and a growing national and international movement of firefighters focused on occupational exposures.

To respond to a lack of documentation of breast cancer rates among San Francisco women firefighters, firefighters Tony Stefani, President of the San Francisco Firefighters Cancer Prevention Foundation, and Anita Paratley, San Francisco Fire Department Battalion Chief and a breast cancer survivor, launched their own department-wide survey. Their survey indicated that breast cancer rates were around six time higher for women firefighters in the department between the ages of 40 and 50 as compared to the national average [31]. Following this, the San Francisco Firefighters Cancer Prevention Foundation and women firefighters collaborated with the San Francisco Department of Public Health to convene a press conference on health threats from toxic smoke produced by building materials and household products. Footage from the press conference was included in the 2013 award-winning HBO documentary “Toxic Hot Seat,” which revealed how chemical companies obscured the public health risks of flame retardants.

The press conference also catalyzed a series of 2012 planning meetings with environmental health advocates and the United Fire Service Women, an organization that advocates for women in the SFFD, with the aim of advancing research on links between occupational exposures and breast cancer risk. Specifically, women firefighters partnered with two environmental health advocacy groups, Breast Cancer Prevention Partners (BCPP) and Commonweal Biomonitoring Resource Center. BCPP is a science-based advocacy organization with a record of helping pass health-protective state and federal legislation to restrict exposures to toxic chemicals. Commonweal is a leader in “advocacy biomonitoring,” or biomonitoring that has the goal of producing evidence of contamination to further policy agendas and increase public awareness of toxics, and had previous experience with firefighter biomonitoring. These advocacy groups connected firefighters to members of the scientific community, specifically environmental health scientist and epidemiologist Rachel Morello-Frosch from the University of California, Berkeley and toxicologist Ruthann Rudel from Silent Spring Institute, a research organization dedicated to uncovering the environmental causes of breast cancer. Both scientists have a long track record with CBPR research. This partnership would evolve into WWBC, with work funded by the California Breast Cancer Research Program [32], the largest state-funded breast cancer research effort in the U.S., the San Francisco Firefighters Cancer Prevention Foundation, and Local 798 of the International Association of Fire Fighters.

The WWBC met frequently to determine study goals and design, and women firefighters were actively involved in recruiting study participants, co-creating exposure assessment interviews, processing blood and urine samples, and drafting recruitment materials and co-authoring publications. The WWBC recruited 86 women firefighters and a demographically similar group of 84 women office workers from the City and County of San Francisco [25]. By including office workers, the WWBC can compare firefighter exposures to women who live and work in the same geographical region but are not involved in firefighting activities, furthering an understanding of which chemicals likely have occupational sources.

As a result of team deliberations, the WWBC adopted an innovative biomonitoring approach. A limitation of traditional biomonitoring studies is that they rely on a priori selection of chemicals for investigation, which may lead to the inclusion of non-relevant chemicals and miss out on important, unanticipated exposures [21]. The study thus expanded methods to include non-targeted analyses. In this case, in addition to looking for specific chemicals of interest, the WWBC screened biological samples for more than 700 chemicals, identifying them by comparing their molecular weights to a curated chemical database. Follow-up measurements were then conducted on a subset of chemicals that were frequently detected in firefighters or relevant to breast cancer etiology [21]. All samples were processed and analyzed at the University of California, San Francisco.

**Fig. 2:**
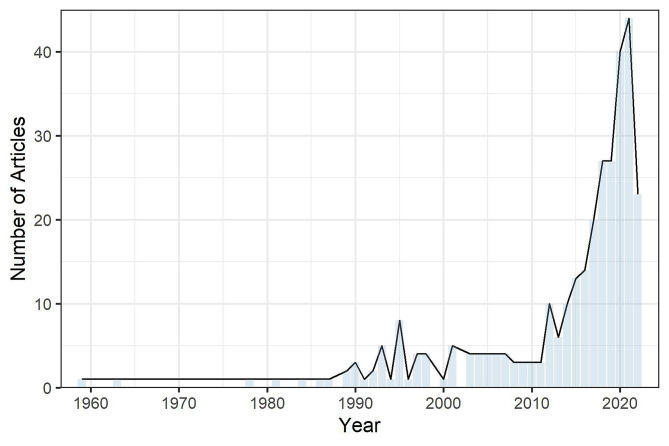
Results from PubMed.gov search using the terms “firefighters” and “cancer” that demonstrates temporal increase in firefighter cancer studies

Peer-reviewed publications on firefighters and cancer began to increase in 2012, around the time of the formation of the WWBC (Fig. 2). The WWBC contributed to this growing number of studies, while responding to the research gap that resulted from the absence of studies on women firefighters and breast cancer. To date, the WWBC has found ubiquitous exposures to PFAS among all study participants, with firefighters having higher concentrations of several PFAS compounds compared to office workers and a representative U.S. sample of similar-aged women [25]. PFAS chemicals were investigated due to their known presence in firefighting foam and equipment such as turnout gear, and links to multiple adverse health outcomes including cancer [33, 34]. Another WWBC study found significant associations between some PFAS and telomere length, particularly among firefighters, which may have implications for carcinogenesis [22]. Additionally, WWBC research has found that firefighters have relatively high exposures to some organophosphate flame retardants [35]. These flame retardants are associated with thyroid disruption in firefighters, which has potential relevance for breast cancer and other adverse health effects [35]. In complementary work, WWBC partner Commonweal, in collaboration with California’s Department of Toxic Substances Control and academic scientists, measured relatively high levels of flame retardants and PFAS compounds in dust collected at fire stations across the U.S. and Canada [36, 37].

To extend into other occupations where women’s workplace exposures are a concern, the partnership won additional funds from CBCRP to add a cohort of nurses and other healthcare workers. While the partnership was initially known as the Women Firefighters Biomonitoring Collaborative (WFBC), it was subsequently renamed to WWBC to reflect this expansion [38]. A key research goal of WWBC now is to develop a biospecimen archive of women workers that is available to other researchers who seek to apply novel analytical approaches to characterize multiple occupational exposures and early biomarkers of effect of relevance for health outcomes such as cancer. The WWBC thus expanded from a community-driven research hypothesis to a project that will create the first women workers’ biospecimen bank.

### Factors contributing to a successful CBPR case

#### Partners have diverse but mutually supportive goals and focus on outcomes at multiple scales

The firefighters, scientists, and advocacy groups that are part of WWBC have different but mutually supportive goals. This led to important outcomes at multiple levels, including impacts on individual behaviors, changes to fire station protocols both locally and nationally, shifts in local, state, and international policies, the development of novel research approaches for studying occupational exposures, and cultural reverberations across the country in the attention paid to the health of women firefighters (Table 1).

Firefighters and partner groups were intent on translating the WWBC’s work into reducing firefighters’ occupational exposures and supporting firefighters diagnosed with breast cancer, even while the study was still ongoing. Impacts from the partnership include upgraded fire station decontamination protocols and a worker training program, improved workers compensation for firefighters diagnosed with breast cancer, and shifts in knowledge and attitudes about the links between the environment and firefighter health. For example, United Fire Service Women leaders drafted a decontamination policy that was adopted in 2019 that ensured firefighting vehicles are outfitted with a decontamination kit. The WWBC also leveraged connections to partners such as the San Francisco Firefighters Cancer Prevention Foundation to promote decontamination protocols that include firefighters continuing to wear self-contained breathing apparatus after a fire is extinguished, dry brushing turnout gear post-fire, and showering and changing clothes immediately after returning from a fire event. As one firefighter who helped launch the study stated,

When I came in it was a badge of honor to have soot all over your face after a fire and your coat to be filthy. And we’d just go back in the firehouse with your dirty turnout pants on and no big deal... We don’t do that anymore, we don’t sleep with our turnout pants next to our bed like we used to.

As another example, BCPP, Commonweal, and firefighters also developed a worker training program, titled “Extinguishing Breast Cancer in the Fire Service.” The training program was implemented in six states, including several trainings in California, to encourage departments across the country to take similar actions to reduce workplace exposures. External collaborations helped shape the training program, with BlueGreen Alliance, an organization that unites U.S. unions and environmental groups, informing the design of the training program using one that had earlier been developed for women steelworkers as a model. In addition, WWBC partners produced two videos about decontamination, one of which went viral within the firefighter community, and organized webinars for firefighters on how to reduce at-home and occupational exposures [39].

Interviewed firefighters repeatedly emphasized how the WWBC contributed to cultural shifts and knowledge about women’s health within the workforce. There was already a burgeoning movement focused on firefighter health in the U.S. and abroad, which WWBC partner The San Francisco Firefighters Cancer Prevention Foundation helped lead. The WWBC, however, ensured that women’s health was no longer “at the fringe,” as an interviewee stated.

Moreover, firefighters, allied advocates, and scientists successfully leveraged study results to help firefighters diagnosed with breast cancer gain increased access to compensation and other benefits offered by presumptive cancer laws (i.e., where illness is automatically considered to be service-related and workers are thus entitled to compensation, medical expense coverage, and medical leave). WWBC participants described the difficulties women firefighters historically faced after being diagnosed, typically having to laboriously recreate their own history of exposures to try and receive compensation. As one San Francisco firefighter stated,

In 2003 [when I was diagnosed], there were no studies. Worker’s compensation is now much more amenable to breast cancer since it’s presumptive [in California]. But there was no research on cancer for women or any kind of reproductive organs... It was really hard to get the women to come forward, because they were afraid of the fire department, they were afraid they were going to run out of time, they didn’t want to deal with workers’ comp.

The WWBC’s research had positive effects beyond San Francisco with respect to the inclusion of breast cancer in presumptive laws. For example, a Massachusetts law that provides paid leave and medical benefits for firefighters with work-related cancer did not initially include breast or reproductive cancers; the law was expanded to include these cancers after Silent Spring Institute scientists shared the WWBC’s research with policymakers [40]. Likewise, starting in 2021, breast cancer was added to the occupational diseases among firefighters eligible for compensation in Colorado [41]. A firefighter interviewee commented that the WWBC influenced this by providing study results and other information to a woman firefighter diagnosed with breast cancer who fought on behalf of the inclusion of breast cancer in the state.

The participation of environmental health advocacy groups with a successful history of research translation and policy change has also been critical for leveraging study results to inform diverse audiences and decision-makers. For example, the WWBC firefighters wanted to change personal behaviors at home; in response, advocacy partner BCPP distributed information about household products as possible sources of harmful exposures and discussed toxic-free alternatives. In addition, BCPP’s involvement in the WWBC supported state and national-level work to better regulate PFAS and flame retardants. This includes the 2020 passage of a bipartisan-approved law in California, SB 1044, that restricts the manufacturing, use, and sale of PFAS-containing firefighting foams. BCPP used the WWBC’s study findings demonstrating elevated levels of PFAS chemicals in women firefighters in their communication on the bill, a coalition sign-on support letter, and their internal FAQs when lobbying legislators. Commonweal and the International Association of Fire Fighters also used the WWBC study data to influence the unanimous approval of a global ban on some PFAS through the United Nations’ legally binding treaty, the Stockholm Convention on Persistent Organic Pollutants.

Partner scientists at the University of California, Berkeley and Silent Spring Institute also have an ambitious plan to address the concerns of the impacted firefighter community, as well as move the biomonitoring field forward by advancing resources and methodologies that increase the capacity to detect exposures to chemicals of concern in other communities. Specifically, the WWBC’s scientists are creating the first archive of biological samples from women workers, to support the advancement of non-targeted biomonitoring approaches, and studying associations between exposures and upstream biomarkers of effect with potential relevance to breast cancer. This can help answer the question of why certain occupations are linked to higher rates of breast cancer and other adverse health outcomes, as well as show the feasibility of novel approaches that improve exposure evaluation.

When asked what contributed to the WWBC’s success, a firefighter commented on the importance of a project with multiple goals and a diverse coalition of partners and external support:

It was multifaceted. It wasn’t just a single study. It had all these different arms of what it was doing. It was much more and caught a lot more people... It helped give us a broader net to capture a lot of interest.

**Table 1:** Impacts of the Women Workers Biomonitoring Collaborative and the scale of action

Impact	Scale of action
Increased knowledge on how to reduce personal exposures to household chemicals through personal report-back and community outreach	Local
Increased knowledge on how to reduce workplace exposures through a worker training program	Local, state
Upgraded fire station decontamination protocols	Local, state
Lent evidence to support firefighter claims that a cancer was occupationally related under cancer presumptive laws	Local, state
Supported adoption of California policy to restrict use of PFAS-containing firefighting foams	State
Shifted cultural attitudes about the importance of addressing women’s health in firefighting	Local, state, national, international
Identified elevated exposures of firefighters to chemicals such as PFAS and flame retardants	Local, state, national, international
Advanced non-targeted biomonitoring methods	National, international
Created a biospecimen archive of women workers	National, international
Supported approval of global ban on some PFAS through Stockholm Convention	International

#### Meaningful communication to participants occurs at all stages of the study

The WWBC has a comprehensive approach to ensuring effective communication to community partners occurs throughout the study. To date, this has included setting early expectations about study goals, adopting novel methods for reporting back personal research results, peer-to-peer communication approaches, and sharing results before publication in peer-reviewed journals.

From the beginning, the partnership communicated realistic expectations for what the study could aspire to achieve. When firefighters were establishing relationships with the WWBC’s scientists, they shared their hopes that a health study would tell them whether occupational exposures were leading to higher breast cancer rates among their workforce. As a result of difficulties with assessing a causal connection between exposures and disease in a small study population, scientists conveyed they could instead investigate whether chemicals of relevance to breast cancer were elevated in firefighters relative to a comparison group of women workers. In addition, they suggested assessing relationships with upstream effect biomarkers of relevance to breast cancer, including thyroid hormone disruption and impacts on telomere length. As one partner scientist emphasized,

We first communicated, we can’t find out why you’re getting breast cancer specifically, but we can find out about what your exposures are like, including notable or unusual exposures that might be related to breast cancer.

In this way, the WWBC designed a meaningful study that addressed community questions about occupational exposures of relevance to breast cancer without overpromising what results would reveal. Furthermore, adopting a biomonitoring study as the research design was important for highlighting opportunities for collective and individual action to reduce exposures of concern.

Moreover, the WWBC has dedicated substantial resources to ensuring that study results are meaningfully shared. Two partners in the WWBC, Silent Spring Institute and Commonweal, contributed their knowledge as national leaders of reporting back personal exposures. In particular, the study employed an interactive web-based tool developed by Silent Spring, the Digital Exposure Report-Back Interface (DERBI), that creates reports that include information about personal exposures, aggregate study results, health outcomes related to the test chemicals, and ways to reduce exposures [42]. An example report is accessible at https://wfbc.reportback.org/wfbc/report/login/demo. The WWBC team conducted multiple focus groups with study participants to ensure that relevant information was included in the digital reports and that figures and text would be useful. Moreover, rather than scientists leading the report-back process, firefighters themselves walked their peers through their DERBI reports to ensure that results were understandable. One firefighter contrasted her experience with the WWBC’s report-back process to a different study that a colleague participated in:

A friend of mine had his blood taken and then asked if I could go with him to get the results at this town hall meeting. And this scientist got up and she handed out sheets with people’s own results on them. And then she started talking and she was not talking to firefighters, she was indecipherable... I remember [my friend] took the sheet of paper with his results and threw it in the trash. And he asked, “Did you understand it?“ And I was like, “No.“ And he’s like, “Me neither.“ None of the scientists we work with [on the WWBC] are like that.

In addition, the WWBC made results available to firefighter and office worker participants in San Francisco before publication in scientific journals through community meetings, webinars, and article preprints available on its website. By doing so, the WWBC ensured that press coverage of study results did not precede individual report-back. This helped avoid “surprises,” as well as feelings among participants that the team was simply extracting data without giving back results in ways that participants could understand and use.

#### The partnership is committed to prevention-based science, including exposure reduction and policy change beginning early in the research process

Given that the extended timeline for producing scientific results often does not match community partners’ desire for more immediate action, the WWBC also supported a direct action component to the work beginning early in the partnership. While the WWBC uses novel scientific approaches to identify exposures not included in targeted methods, emphasizes chemicals linked to breast cancer, and is the first to focus on women firefighters, there was already-existing scientific evidence that firefighters have elevated exposures to chemicals of concern and higher rates of certain cancers. This warranted taking early action to ensure that study participants and coworkers benefited from the existing body of evidence even as new knowledge was generated by the WWBC.

Firefighters, with support from advocacy and scientist partners, thus launched the previously described national training program on reducing workplace exposures, produced informational videos, and made changes to fire station decontamination protocols before WWBC study results were completed. As a partner scientist stated,

The cool thing about this collaborative is that a lot of the work has been happening in parallel. It wasn’t like we were going to wait until all of the data were in before people started doing great things. So, there’s a lot of independence and initiative among members of this group to take on projects and to lead them…We don’t want to wait around for the data to roll in, people struck while the iron was hot.

Similarly, a WWBC firefighter stated,

The training was very important because it gave us a way to have an impact while we were waiting for the results. I’m not very patient, and I don’t think many firefighters are, because we’re used to just doing, doing... I know now how important the science is, but the education is really where the boots-on-the-ground changes are being made.

Moreover, firefighter leaders in the WWBC, such as Heather Buren, chair of the San Francisco’s United Fire Service Women, and Tony Stefani, president of the San Francisco Firefighters Cancer Prevention Foundation, lobbied for policy changes in Sacramento, CA, and Washington, D.C. throughout the CBPR partnership. For example, Stefani gave testimony for a new federal bill on toxics that ultimately passed. One firefighter commented, “it was so heartening that even without study results, they started changing policies and systems.”

Overall, a key element of the WWBC is the commitment of partners to prevention-based science that does not require high statistical certainty and fully established cause and effect relationships, but rather leads to swift action to reduce exposures in the face of existing and strong evidence of harm. Partner scientists and advocates in the WWBC all have a long history of being dedicated to prevention-based science. For example, Silent Spring Institute and BCPP are both dedicated to breast cancer prevention and Commonweal helps lead biomonitoring studies that promote policy agendas to prevent pollution. In an example that illustrates a participating researcher’s commitment to prevention-based science, several firefighters discussed how Dr. Morello-Frosch argued that breast cancer among women firefighters was a problem regardless of whether or not rates were statistically elevated within their small population. As one firefighter stated,

One of the scientists stated, “Why do you care about how many? I don’t care about the percentage in your department, I care about if they all got it before menopause…That’s not typical…You’re a relatively healthy population.”

#### Study emphasizes collaborative partnerships and co-learning in knowledge production

Promoting opportunities for co-learning and integrating firefighter expertise, skills, and resources into the partnership was critical for the WWBC’s success. The equal partnership ethos is reflected in the research leadership approach: firefighters Stefani and Buren act as co-principal investigators on the grant funding the project. Firefighters, scientists, and advocates in the WWBC all emphasized the importance of reciprocal knowledge and skills transfer among research partners. Firefighters in particular have been central to all aspects of the scientific process, including recruitment, development of the exposure assessment survey, preparation of specimens for analysis, and dissemination of results. Firefighters also collaborated in writing peer-reviewed articles, and it was the firefighters who initiated the study and selected partner scientists and advocates.

A WWBC lead scientist underscored the contributions of firefighters’ expertise to the research:

Without this collaboration, none of this scientific work would be possible. There’s no way I could go to a firehouse and introduce myself and invite people to take part in a study…This collaboration ensures that the science is rigorous, in addition to making sure that it produces the necessary evidence to make change in policies and laws.

Firefighter leaders not only recruited participants from within their ranks, but the comparison group of city workers was made possible in part because the then-chief of the San Francisco Fire Department wrote a letter to all city employees requesting their participation in a study to benefit frontline workers.

Furthermore, as one BCPP scientist stated, meaningful participation by firefighters gave the research team “a sense of the culture, leadership, and schedules, which were just as important as the chemical exposure data itself.” A firefighter corroborated this, saying

We met with these scientists and looked through [the questionnaires] and I’m like, “No, we have to use our language. You’ve got to say apparatus bay. It’s not the place where you park the rigs.” If it’s not right then the people taking that questionnaire are going to be like, “I don’t trust these people.” And I was told by the scientists, “This is why this is so important that the community is involved in this. Otherwise, this would be a foreign language to us.” But now [the scientists] could have a conversation with any firefighter…they’ve learned another language..

Similarly, firefighters became familiar with scientific terminology and methods to guide colleagues in interpreting their personal exposure reports, as well as present the study to national women’s firefighter groups and fire departments across the country. As one scientist described her interactions with a firefighter who presented the WWBC’s results at a major firefighting conference:

She spent so much time going over it with me, asking questions, making sure she got the science right. She emphasized to her peers that if I can understand this then you all can as well. She brought her lived experience as a firefighter and breast cancer survivor to the scientific results.

In another demonstration of how the study allowed community partners to gain research skills and confidence in their ability to engage the science, several firefighters positively reflected on their experience helping process samples in the lab. As one stated, “We’re in lab coats and I’m like, ‘This is crazy. I can do this.’”

#### Study builds strong relationships among partners, including outside of formal research settings

The WWBC spends time cultivating strong personal connections and mutual respect among partners both within and outside of formal research settings. This has included through retreats, cross-country travel to attend firefighter conferences together, dinners together, and scientists attending community events.

Moreover, WWBC participants repeatedly commented on how the collaboration is supportive and respectful. As one firefighter stated, “I was one person feeling pretty helpless… Scientists had the compassion to listen… But they also gave us some teeth to cause change.” One scientist recalled how her early interactions with firefighters were defined by their distress with coworkers getting premenopausal breast cancer, particularly given how athletic and young they were. As a result of the collaboration, a firefighter conveyed to her that she could now tell her co-workers “help is on the way,” which is what firefighters tell distraught members of the public. Many participants argued that the all-women partnership contributed positively to the collaboration. As one stated, “it was a bunch of professional women who had a lot of respect for other professional women and understood how it is in a male-dominated field.”

Furthermore, the WWBC participants often remarked on how partners are committed to the “long haul.” The WWBC has been ongoing for over a decade, with new questions continually being investigated and the study has expanded to include new partnerships such as with healthcare workers.

#### Institutional systems that support CBPR are in place

Another factor critical to the success of the WWBC is funding from the California Breast Cancer Research Program (CBCRP), which specifically supports CBPR. CBCRP was created when breast cancer advocates won passage of statewide legislation in 1993 to fund innovative breast cancer research. As part of their funding profile, CBCRP awards grants to support partnerships between communities affected by breast cancer and experienced research scientists. As a result of this CBPR funding source, firefighters were able to combine their specialized knowledge and interests with the expertise and resources of established scientists and environmental health advocates to address community-driven questions. In addition to funding researcher-community partnerships, CBCRP involves advocates in every aspect of decision-making, including program planning and grant application review, and runs matchmaking and training sessions to connect affected communities with interested scientists and prepare them for success.

Moreover, collaborating with labor unions and the City of San Francisco helped with many aspects of the CBPR initiative, including recruiting participants and translating results. For example, in addition to support among firefighters’ leadership, city employees, including leadership at the San Francisco Department of the Environment, helped recruit office workers for a comparison group. The union and outside foundations, such as the San Francisco Firefighters Cancer Prevention Foundation contributed funding and other resources. Furthermore, the fire chief at the time allowed San Francisco Fire Department employees to participate in the study while on duty, and gave individuals who were on desk detail, because of an injury or pregnancy, the option to help with tasks such as recruiting participants and processing biospecimen samples. This is in stark contrast to most other occupational settings where there are typically institutional barriers to studying worker exposures. As a WWBC scientist stated, “it’s typically extremely difficult to do occupational health studies in the U.S. Companies won’t let you in, workers aren’t organized. Even NIOSH [The National Institute for Occupational Safety and Health] has trouble getting in.”

## Discussion

The WWBC shares characteristics with other successful CBPR partnerships, including cultivating shared governance in all phases of the research process, democratizing knowledge production, developing meaningful communication to all partners and study participants, building on strengths and resources within the community, increasing community capacity to respond to environmental health problems, cultivating long-term commitments by all partners, and influencing policy, regulatory, and organizational agendas [3, 43–45]. The WWBC was initiated by firefighters, who have played a lead role in formulating the research questions and study design, collecting and analyzing data, co-authoring peer-reviewed articles, communicating findings to their peers, and translating results into community and policy-level actions. Other CBPR literature has emphasized the importance of leveraging community knowledge [46, 47], and the WWBC exemplifies an exceptional collaboration of highly specialized knowledge from scientists and firefighters.

Underscoring the successful experience of the WWBC within a CBPR framework, the WWBC participated in the Measurement Approaches to Partnership Success (MAPS) study. MAPS is a survey project by leading CBPR researchers from the University of Michigan and representatives from multiple Detroit organizations that is aimed at advancing understanding of success in long-standing CBPR partnerships and developing a validated tool for self-evaluation [48, 49]. The WWBC scored highly in all seven thematic areas that the MAPS project identified as critical to successful CBPR, including realization of benefits over time, partnership equity, building enhanced capacity among partners, and sustainability. Thus, in addition to the strengths of the WWBC highlighted by those who participated in qualitative interviews and discussions for this article, the WWBC was a leading example of CBPR in an evaluation conducted by an external team of researchers.

While the WWBC is indebted to long-established CBPR practices, the collaborative also advances approaches that strengthen the ability for CBPR to affect change at multiple levels. In particular, future CBPR can consider whether it would be useful to integrate additional partners with particular skills into the work beyond the traditional community-scientist dyad, such as experienced advocacy groups who can facilitate policy and organizing impacts. While in other contexts it might be appropriate to keep partnerships limited [50], a diverse coalition of study partners and external supporters has been critical for achieving the WWBC’s multi-scalar goals. By involving workers, scientists, environmental health advocates, private foundations, unions, and government staff, the WWBC impacts are broad reaching and include shifts in individual behaviors, changes to fire station protocols across the country, the adoption of stringent environmental health and occupational laws, advances in environmental health research methodologies, and an increased focus on women workers’ health. It was firefighter connections, including with the International Association of Firefighters and advocacy groups, that facilitated U.S.-based and international policy impacts. As another example, support from unions and city government staff was critical for the successful study recruitment of both firefighters and a comparison group of city workers.

An additional benefit of conducting CBPR with a broader array of research partners is that it potentially enhances the sustainability and resilience of the project. For example, academic researchers’ time and resources can be highly tied to institutional obligations and support, and academia is not always understanding of CBPR projects or willing to support them financially. In this case, supporting firefighter groups, including The San Francisco Firefighter Cancer Prevention Foundation, The United Fire Service Women, and The International Association of Firefighters-Local 798, lent funding, in addition to aforementioned staff time to the project.

Other partners that CBPR projects can consider include policymakers, physicians, legal experts, and local businesses [45]. Given that a traditional community-academic model is not always effective and that including other partners (such as professional advocacy groups and unions) can help elevate impact, it’s important that funding programs that support CBPR keep these alternative models in mind.

Furthermore, the WWBC leveraged existing scientific evidence to ensure the work translated into individual, community-wide, and policy impacts beginning early in the collaboration. The study was explicitly designed so that advocacy groups and researchers could support early action based on current scientific knowledge and prevention rather than make community partners wait until results were finalized. This addresses communities’ urgent need for more rapid change than typifies the typical timescales of scientific knowledge production.

Ensuring meaningful communication to community partners and study participants is an established CBPR principle, and the WWBC exemplifies and extends this approach in multiple ways. Scientist partners set early expectations about what a study could and could not achieve. Moreover, research translation was assisted by the involvement of national leaders on report-back, namely Silent Spring Institute and Commonweal. Environmental health researchers are often reluctant to communicate personal biomonitoring results due to a lack of resources and training, and concerns about participant worry [51]. CBPR, however, favors a “right-to-know” approach, which includes translating personal research results to study participants and sharing overall results with community stakeholders and policymakers so they can take action [2, 52, 53]. The WWBC leveraged Silent Spring’s digital report-back tool, which enables the production of high-quality, interactive reports to share individual participant results. In this respect, preexisting resources and expertise of partners contributed to the capacity to carry out meaningful translation work.

Moreover, scientists worked with firefighter study leaders to train them in sharing personal exposure reports; having peer-to-peer research translation increases the scientific expertise of community leaders and facilitates other study participants’ connection to the study and ability to interpret their results. Finally, study results were shared with participants frequently throughout the project and before final publication in academic journals. Other CBPR projects can look towards successful models of research translation such as this to ensure data is shared effectively with participants. Critically, the WWBC’s dissemination and translation work, including support for returning personal results, was built into the project’s budget from the start.

While much of the CBPR literature focuses on the importance of factors internal to the partnerships, greater attention needs to be paid to the institutional factors that support these types of research partnerships. Despite the importance of preexisting resources and expertise among study partners, the WWBC’s successes have been highly dependent on external structural factors. This includes funding structures that enable projects to achieve deliverables, while also allowing for nimbleness to respond to emerging science-policy opportunities, and worker support systems.

The WWBC achievements are facilitated by California’s unique funding program for breast cancer research, the CBCRP, which supports researcher- community partnerships. Through this program, there is funding earmarked for pilot projects so that research approaches are developed collaboratively from the very beginning and the inclusion of advocates in the grant review process ensures projects are responsive to evolving community and policy-relevant issues. While federal and private foundation support of CBPR has substantially increased since 1996, when the National Institute of Environmental Health Sciences began supporting action-oriented partnerships, funding opportunities of such research should be augmented [54, 55]. Funding initiatives can focus on planning grants that help set priorities and create the relationships needed for long-term CBPR partnerships, long-range funding to support the extended commitments required for effecting change, and infrastructure grants (for example, to hire program staff; [54]). Government and private funders can be encouraged to support more CBPR in their research portfolios not only for the impacts on community health and policy, but also because of improvements to the rigor, relevance, and reach of the scientific enterprise itself [2]. In this case, firefighter and advocate involvement in scientific research strengthened lines of inquiry and study protocols, made the work more relevant to the lives of women firefighters, and increased its reach in the community and policy arenas.

Moreover, institutional structures such as a unionized workforce and supportive leadership, within the firefighting force and at city hall, contributed to both a successful study and its resulting policy impacts. As described, firefighter study recruitment was aided by connections to firefighter organizations such as United Fire Service Women and Local 798 of the International Association of Fire Fighters, and female office employees were recruited with help from the City and County of San Francisco. In contrast, occupational health research can be difficult or impossible to conduct in situations where workers are concerned about job security or when employers are not cooperative in providing access to the workplace. Occupational health research can be challenging even for entities with the legal authority to conduct such research such as the National Institute for Occupational Safety and Health (personal communication from a former National Institute for Occupational Safety & Health epidemiologist). It is thus important for supporters of CBPR to not just focus on factors internal to research partnerships, but also critically examine and advocate for the institutional factors that can better support this research in the first place.

### Limitations

A limitation to our qualitative study is that it relies on a small sample of interviews with WWBC leaders and thus does not encompass the perspectives of a broad range of actors who are impacted by this collaborative research. The article, however, is co-authored by WWBC firefighters, scientists, and advocates and is meant to be reflective of the experiences and knowledge of outcomes of those most intimately involved in this partnership.

Results may not be generalizable to less-resourced settings. Firefighters are a well-resourced group relative to many other community partners, and other CBPR projects will likely need to be responsive to the needs of partners who are not similarly situated. For example, firefighters in the WWBC are salaried and it was possible for them to dedicate in-kind time to study activities, such as recruiting and scheduling participants. Many other CBPR projects involve communities that are economically marginalized and funding applications should include adequate compensation to enable community participation. The WWBC also received financial support and other resources from government agencies, unions, firefighter organizations, and foundations. Furthermore, firefighters have politically powerful unions and associations and enjoy strong public support, which facilitates their ability to translate research findings into action. Other impacted worker communities, for example, rurally isolated, non-documented agricultural workers, have less political clout and limited media attention as a result of intersecting axes of oppression, including racism, geographical isolation, language barriers, and lack of legal status [56]. Other CBPR projects should formulate strategies to help strengthen communities’ access to resources beyond just financial ones, including media and policy attention. They should also identify and address unequal power and privilege relations between researchers and communities [57].

## Conclusion

The WWBC offers a model that builds on established CBPR practices, but also offers insights into how to increase scientific and policy impacts. Key drivers of the success of the WWBC include incorporating additional partners beyond the traditional community-academic dyad, translating scientific evidence into benefits for community health and policy change early in the partnership, and adopting meaningful communication among partners including training in reporting back personal results. Additional external factors benefited the partnership, including the availability of funding that promotes CBPR, and institutional support for research including a unionized workforce and responsive employers.

## Data Availability

Interview questions available upon request.

## Abbreviations

CBCRP: California Breast Cancer Research Program
CBPR: Community-based participatory research
DERBI: The Digital Exposure Report-Back Interface
MAPS: Measurement Approaches to Partnership Success
NIOSH: National Institute for Occupational Safety and Health
PFAS: Per- and polyfluoroalkyl substances
SFFD: San Francisco Fire Department
WFBC: Women Firefighters Biomonitoring Collaborative
WWBC: Women Worker Biomonitoring Collaborative
